# Complement C4 induces regulatory T cells differentiation through dendritic cell in systemic lupus erythematosus

**DOI:** 10.1186/s13578-015-0052-8

**Published:** 2015-12-23

**Authors:** Hong-Bin Cheng, Rong-Yi Chen, Jing-Ping Wu, Li Chen, Yan-Hua Liang, Hai-Feng Pan, Zi-Feng Pan, Qing-Hua Zhang, Qing Li, Tian-Xi Du, Yong-Mei Lv, Jian-Qiang Shi

**Affiliations:** Teaching Hospital of Chengdu University of TCM, Chengdu, 610072 Sichuan China; Department of Dermatology, Affiliated Hospital of Guangdong Medical College, No.57 Peoples Avenue South, Zhanjiang, 524001 Guangdong China; Laboratory Animal Center, Anhui Medical University, Hefei, 230032 Anhui China; Department of Dermatology, Nanfang Hospital, Southern Medical University, Guangzhou, 510515 Guangdong China; Department of Epidemiology and Biostatistics, School of Public Health, Anhui Medical University, Hefei, 230032 Anhui China; Department of Dermatology, The Second Affiliated Hospital, Anhui Medical University Hefei, 678 Furong Road, Hefei, 230601 Anhui China

**Keywords:** Complement C4, Systemic lupus erythematosus, Treg cells, TGF-β

## Abstract

**Background:**

Systemic lupus erythematosus (SLE) is a prototypic systemic autoimmune disease. Complement component 4 (C4) has be proved to play a role in pathogenesis of SLE. In the present study, we investigated the effect of C4 on T cells differentiation.

**Methods:**

Thirty SLE patients were included in this study. CD4+ T cells were isolated from healthy subjects, and dendritic cells (DCs) were isolated from healthy subjects or SLE patients. C4 was supplemented to co-incubate with T cells and DCs.

**Results:**

Serum C4 concentration was positively correlated with regulatory T cell (Treg) percentage (R^2^ = 0.5907, p < 0.001) and TGFβ concentration (R^2^ = 0.5641, p < 0.001) in SLE patients. Different concentrations of C4 had no effect on T cells differentiation. Co-incubated T cells with DCs and C4 for 7 days, the Treg percentage and TGF-β concentration were significantly elevated. In addition, pre-treated DCs (from healthy subjects or SLE patients) with C4 and then co-incubated with T cells, the increases of Treg percentage and TGF-β concentration were also observed.

**Conclusion:**

C4 takes part in T cells differentiation to Treg cells via DCs.

## Background

Systemic lupus erythematosus (SLE), a severe multisystem autoimmune disease, is characterized by a loss of immune tolerance to self-antigens (Ags), which results in the persistent production of pathogenic autoantibodies, activation of lymphocyte and release of inflammatory mediators [1]. Many factors including genetic, environmental and hormonal contribute to initiate and drive SLE pathogenesis [2]. However, the exact pathogenic mechanisms remain to be elucidated.

*Regulatory T cells (Tregs)* are involved in inhibition process of CD4+ Th cells activation, cytotoxic CD8+ T cells differentiation and B cell activation [3–5]. The definitive role of Treg cells in SLE remains not fully clear. In SLE patients, the reduced number and function of CD4+CD25 Foxp3 Tregs have been observed [6]. The deficiencies of Tregs might contribute to the breakdown of self-tolerance and the development of the autoimmune response in SLE patients [7].

Complement component 4 (C4), a protein involved in complement system, takes part in multiple aspects of the immune system [8]. It has also be shown that complement system is implicated in pathogenesis of SLE and has important protective functions in SLE [9, 10]. SLE patients have decreased C4 copy number and serum C4 concentration [11], and low copy number of C4 is identified to be a risk factor for SLE susceptibility [12]. The mechanisms of the link between abnormal complement system and SLE or SLE-like diseases have been partly proposed, including impaired clearance of immune complexes and apoptotic cells, abnormal development of B cell self-tolerance [8, 13]. In a recent study, it was shown that knockout C4 complement gene mice had a decreased frequency of Treg cells [14], which implies that C4 complement can promote differentiation of T lymphocyte to Tregs. C4-derived peptide has been demonstrated to inhibit Th1 cytokine production [15]. However, very few literatures have been emerging to elaborate the effects of C4 on Treg cells. Transforming growth factor-beta (TGF-β) is critical for the development and differentiation of Tregs, and it is an important indicator for reflecting the function and stabilization of Tregs [16]. In present study, we investigated whether C4 participated in T cells differentiation and the potential mechanism was also explored.

## Methods

### Subjects

Thirty SLE patients, with 10 female and 20 male, regularly followed up at the Second Affiliated Hospital of Anhui Medical University (from November 2012 to June 2014) were included in this study. The human study was approved by Ethics Committee of the Anhui Medical University and written informed consents were obtained from all patients. The clinical information including age, gender, duration of disease and concentrations of α-globulin, β-globulin, IgG, IgA and IgM were collected. The concentrations of α-globulin, β-globulin, IgG, IgA and IgM were detected.

### Isolation and culture of T cells

The T cells were isolated from peripheral blood mononuclear cells (PBMCs) of healthy subjects. PBMCs were isolated by Ficoll density gradient centrifugation and collected to culture in RPMI1640 medium (Gibco, USA). Immuno-magnetic beads (Miltenyi Biotec, Germany) were used to isolate CD4+ T cells according to the manufacturer’s instructions. The obtained T cells were cultured in RPIM1640 medium containing 10 % fetal bovine serum (Gibco, USA), penicillin–streptomycin (100 IU/ml and 100 μg/ml, Sigma, USA) and 2 mM l-glutamine (Sigma, USA). In addition, different concentrations (0, 250, 500 and 1000 μg/ml) of C4 were supplemented in medium to co-incubate with T cells for 7 days.

### Isolation and culture of dendritic cells

The dendritic cells (DCs) were also isolated from peripheral blood of healthy subjects or SLE patients with C4 number <4 copies. Mononuclear cells were collected from the blood supernatant. RPMI1640 medium, containing 10 % autoserum (Gibco, USA) and 50 ng/ml rhIL-7 (R&D Systems, USA), was used to culture and induce differentiation of mononuclear cells for 24 h. In some experiments, 500 μg/ml complement C4 (Sigma-Aldrich, USA) was pre-treated with DCs for 48 h. Then C4 treated DCs were co-cultured with 2 × 10^4^ freshly isolated CD4+ T cells in round-bottom 96-well culture plates for 7 days. In other experiments, DCs co-incubated with 2 × 10^4^ freshly isolated CD4+ T cells in RPMI1640 medium containing 500 μg/ml complement C4 for 7 days.

### Detection of serum complement C4 and TGFβ concentrations

The isolated serum from patients was stored at 80 °C. The serum C4 level was measured using a human ELISA kit (DRG-international, Germany). The TGF-β concentrations were also determined using commercial ELISA kit (R&D Systems, USA). All the experimental operations were performed according to the manufacturer’s instructions.

### Flow cytometry

T lymphocyte subsets were analyzed by flow cytometry (Beckman Coulter, USA). The cells were stained using PerCP-Cy5.5-labeled anti-human CD3 (eBioscience, USA) and fluorescein isothiocyanate (FITC)-labeled anti-human CD8 (eBioscience, USA). Treg cells were identified using FITC-labeled anti-human CD4 (eBioscience, USA). The various T lymphocyte subsets were calculated using the antibody signals of the specific proteins.

### Real-time quantitative PCR

FlexiGene DNA Kit (Qiagen, Germany) was used to extract genomic DNA from human whole blood. TaqMan^®^ Genotyping Master Mix (Applied Biosystems, USA) was used to quantify gene expression, with RNaseP (Applied Biosystems, USA) serving as reference genome. The C4 copy number was analyzed by CopyCaller™ Software.

The total mRNA of T cells was extracted with RNeasy kit (Invitrogen, USA). cDNA was synthesized using ImProm-II™ Reverse Transcription System (Promega, USA). The mRNA expression of FOXP3 was quantified by Applied Biosystems 7500 Real-Time PCR System (Applied Biosystems, USA) with Power SYBR^®^ Green PCR Master Mix (Applied Biosystems, USA). The FOXP3 primers are as follows: forward 5′-AGGCTTCATCTGTGGCATCAT-3′; reverse 5′-CTTGCGGAACTCCAGCTCAT-3′. β-actin serves as the control gene to normalize the quantification of FOXP3. The primers of β-actin are as follows: forward 5′-CTCCATCCTGGCCTCGCTGT-3′; reverse 5′-GCTGTCACCTTCACCGTTCC-3′. $\Delta \Delta^{{{\text{C}}_{\text{t}} }}$ method was used to analyze the relative gene expression.

### Statistical method

The results are given as mean ± standard error (SEM). Statistical significance among three experimental groups was assessed by ANOVO, and the difference between two experimental groups was assessed by independent-samples T test. Correlations between plasma complement C4 and Treg percentage/TGF-β were determined by Pearson correlation coefficient. All statistical analyses were performed using the SPSS 17.0. Significance was assumed at a p level of less than 5 %.

## Results

### Summary of study group characteristics and observed expressions

As shown in Table 1, the patients with C4 copy numbers <4 were matched with the patients with C4 copy numbers = 4 or C4 copy numbers >4 for sample number, and there were no significant differences in age, gender and duration of disease among three groups. The levels of α-globulin and γ-globulin in subjects with C4 copy numbers >4 were significantly decreased compared with subjects with C4 copy numbers <4. The concentrations of IgG, IgA and IgM were decreased with the increasing of C4 copy numbers. In addition, serum complement C4 concentrations were elevated with the increasing of C4 copy numbers.

**Table 1:** Summary of study group characteristics and observed expressions

Variable	C4 copy numbers <4	C4 copy numbers = 4	C4 copy numbers >4
Number of samples (n)	10	10	10
Age (years)	52.4 ± 6.8	53.3 ± 4.9	51.8 ± 8.4
Gender, male/female (n)	7/3	6/4	7/3
Duration of disease (years)	3.5 ± 1.3	2.9 ± 0.9	2.7 ± 1.5
α-Globulin (g/cl)	0.72 ± 0.07	0.69 ± 0.05	0.59 ± 0.06*^#^
γ-Globulin (g/cl)	0.82 ± 0.11	0.71 ± 0.09*	0.70 ± 0.07*
IgG (mg/cl)	1459 ± 104	1302 ± 58*	1219 ± 49*^#^
IgA (mg/cl)	31.8 ± 4.9	26.5 ± 6.1*	20.6 ± 3.9*^#^
IgM (mg/cl)	32.5 ± 5.3	25.2 ± 4.18*	19.4 ± 2.0*^#^
Serum complement C4 (g/L)	49.3 ± 7.9	78.4 ± 12.3*	110.3 ± 15.2*^#^

Data are presented as the mean ± standard error of mean

*Ig* immunoglobulin

* p < 0.05, vs group (C4 copy numbers <4)

^#^p < 0.05, vs group (C4 copy numbers = 4)

### Correlation of serum complement C4 concentration and Treg percentage

**Fig. 1:**
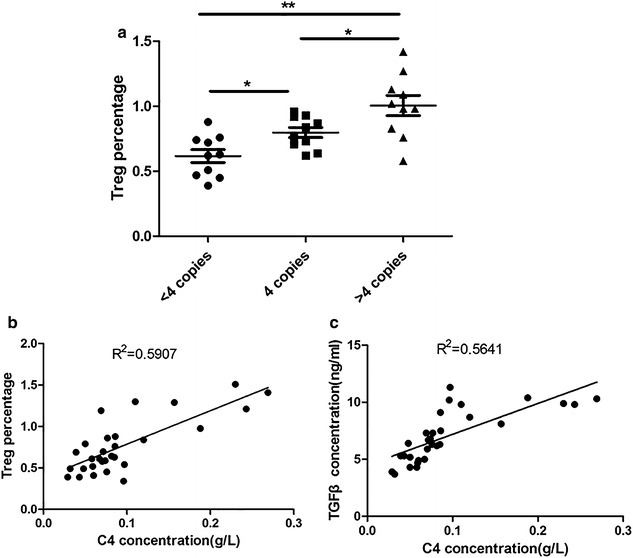
The correlation between serum complement C4 concentration and Treg percentage. **a** The Treg percentage was detected by flow cytometry; n = 10; the data were expressed as mean ± standard error; *p < 0.05; **p < 0.01. **b** Spearman correlation was used to evaluate the relationship between Treg percentage and C4 concentration. **c** Spearman correlation was used to evaluate the relationship between Treg percentage and TGFβ concentration

The serum Treg percentages of SLE patients were detected. As shown in Fig. 1a, the values of Treg percentage in patients with C4 < 4 copies, = 4 copies and >4 copies respectively were 0.62, 0.79 and 1.00. The Fig. 1b, c showed that peripheral serum C4 concentration was positively correlated with Treg percentage (R^2^ = 0.5907, p < 0.001) and TGFβ concentration (R^2^ = 0.5641, p < 0.001) respectively in SLE patients.

### The effect of complement C4 on differentiation of T lymphocytes

**Fig. 2:**
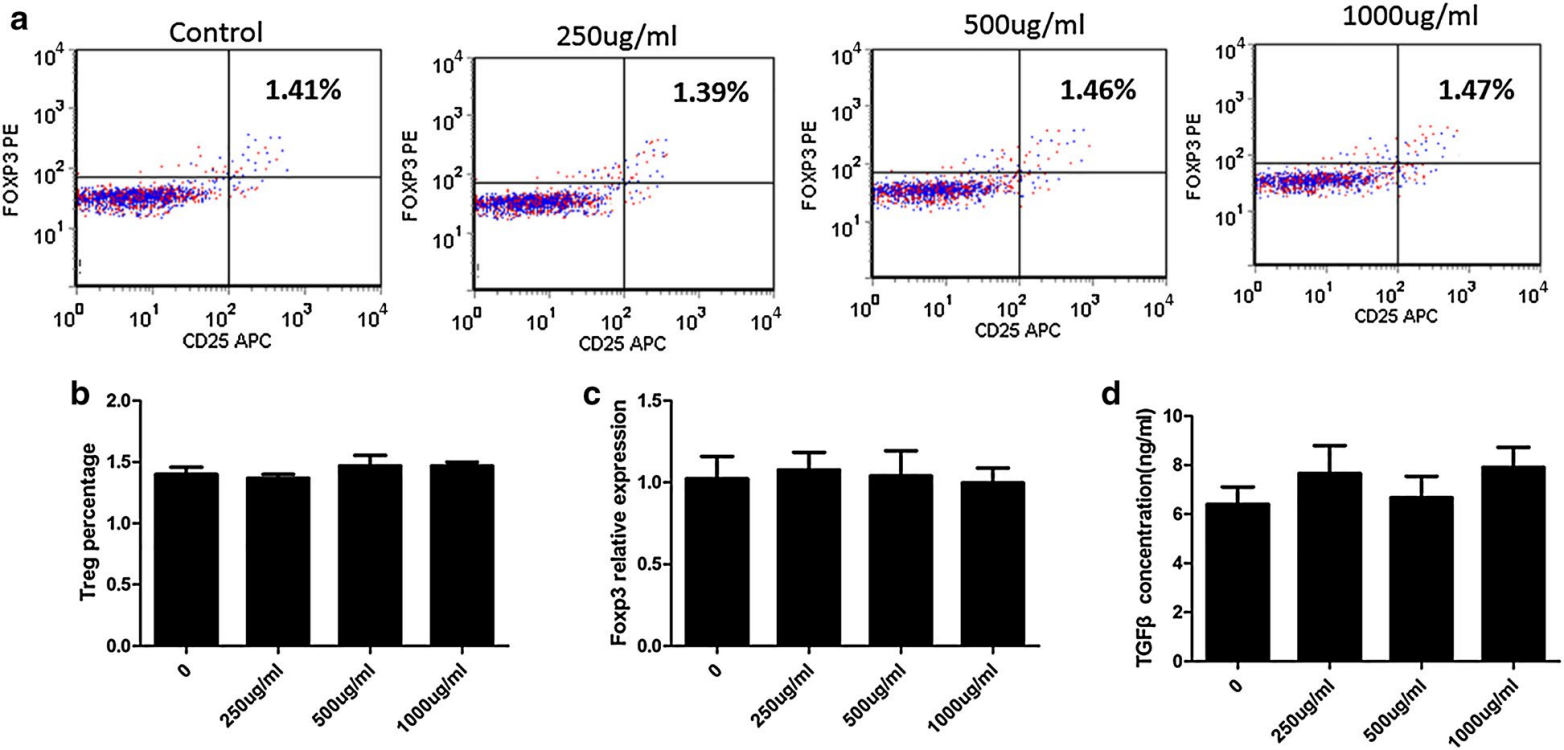
The effect of complement C4 on differentiation of T lymphocytes. **a** Flow Cytometer was used to determine the CD4+CD25+FOXP3+Treg percentage. **b** The statistical result of Flow Cytometer was shown. **c** Real-time PCR was used to quantify Foxp3 relative mRNA expression. **d** TGFβ concentration of cells was determined by ELISA kit

Administrated T cells with complement C4 of different concentrations (DC:T = 1: 6) for 7 days, the CD4+CD25+FOXP3+Treg percentage was determined by Flow Cytometer. The results showed that there was no difference in CD4+CD25+FOXP3+Treg percentage between cells with or without complement C4 treatment (Fig. 2a, b). The differences of Foxp3 mRNA expression and TGFβ concentrations were not observed in cells treated with complement C4 of different dosages (Fig. 2c, d).

### Complement C4 promotes T lymphocytes differentiation via DCs in healthy subjects

**Fig. 3:**
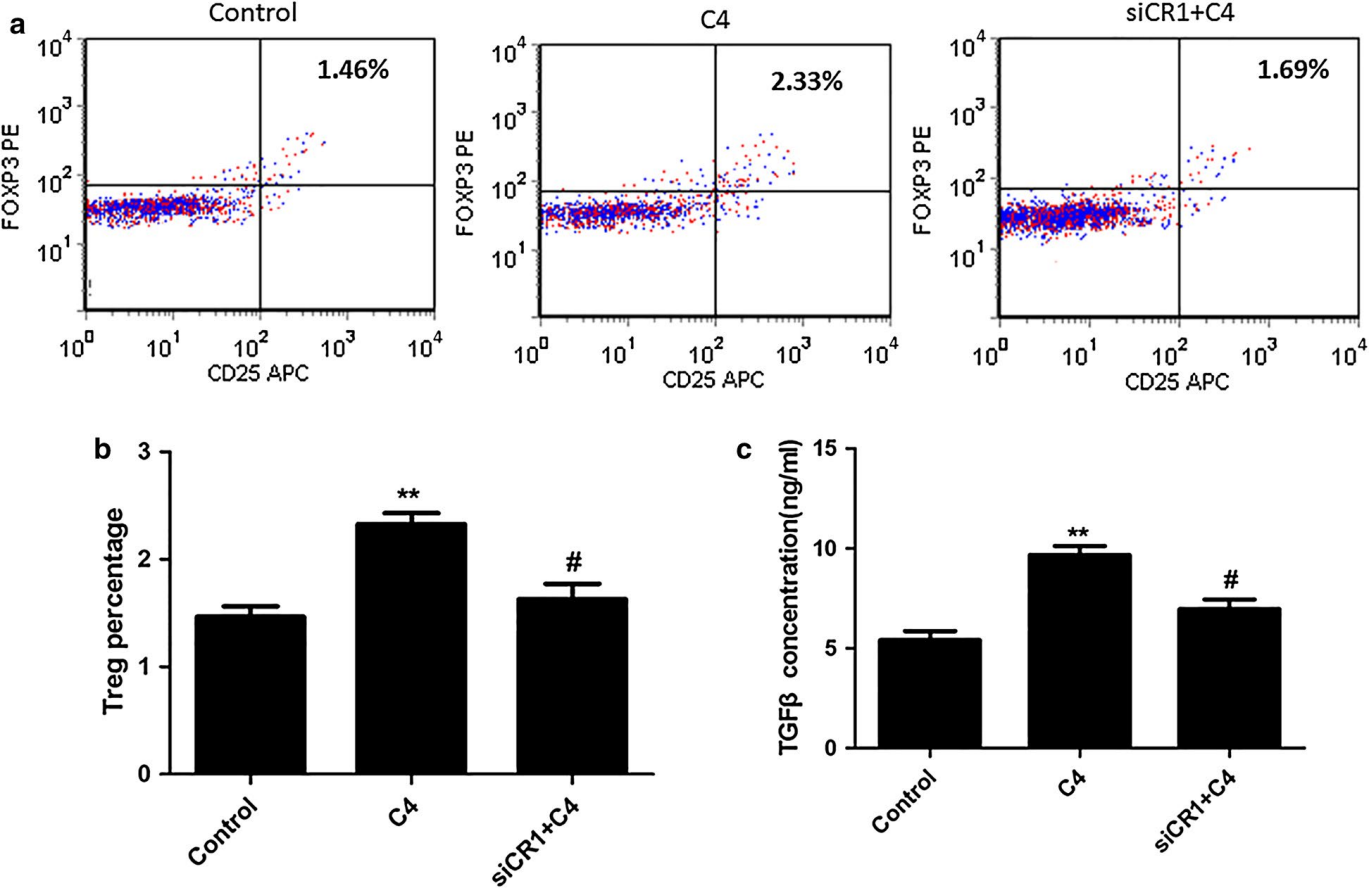
Complement C4 promotes T lymphocytes differentiation via DCs. Pre-incubated T lymphocytes with DCs for 48 h, and then 500 μg/ml complement C4 was supplemented for 7 days. **a** Flow Cytometer was used to determine the CD4+CD25+FOXP3+Treg percentage. **b** The statistical result of Flow Cytometer was shown. **c** TGFβ concentration of cells was determined by ELISA kit. **p < 0.01 vs control; ^#^p < 0.05 vs C4 group

Isolated DCs from healthy subjects. Pre-incubated T lymphocytes with the DCs for 24 h, and then supplemented with 500 μg/ml complement C4 for 7 days. The results of Flow Cytometer showed that complement C4 significantly increased CD4+CD25+FOXP3+Treg percentage in cells pretreated with DCs. However, the effect was reversed by interference of CR1, a receptor of complement C4 (Fig. 3a, b). The concentration of TGFβ was also increased by complement C4 in T lymphocytes pre-incubated with DCs, and supplement of siRNA-CR1 decreased TGFβ concentration.

**Fig. 4:**
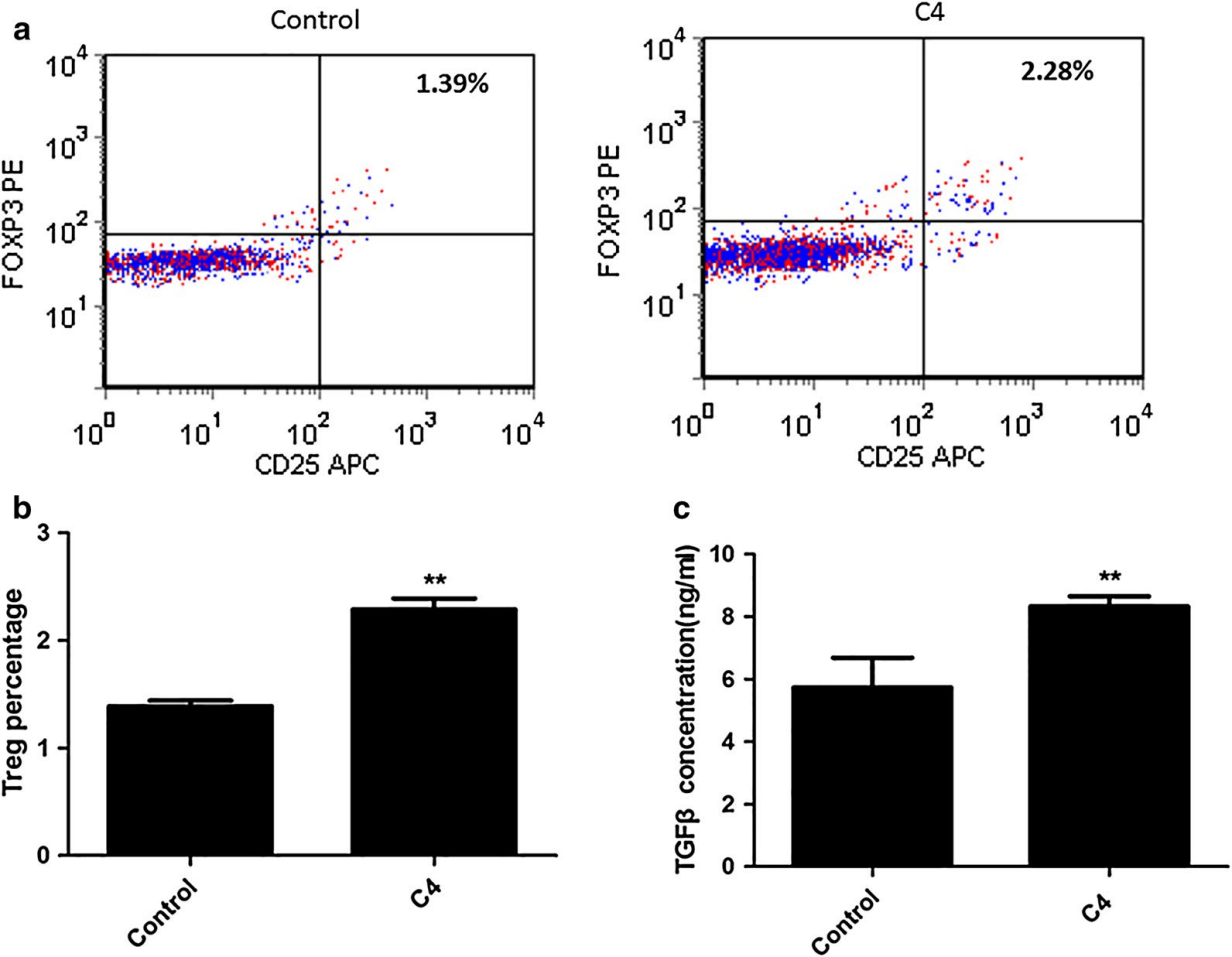
Pretreating DCs from healthy subjects with complement C4 promotes T lymphocytes differentiation. Pre-incubated DCs (from healthy subjects) with 500 μg/ml complement C4 for 48 h, and then incubated with T lymphocytes for 7 days. **a** Flow Cytometer was used to determine the CD4+CD25+FOXP3+Treg percentage. **b** The statistical result of Flow Cytometer was shown. **c** TGFβ concentration of cells was determined by ELISA kit; **p < 0.01 vs control

On the other hand, treated DCs (from healthy subjects) with 500 μg/ml complement C4 for 48 h, and then incubated with T lymphocytes for another 7 days. The data also shown that CD4+CD25+FOXP3+Treg cells percentage (Fig. 4a, b) and TGFβ concentration (Fig. 4c) were markedly elevated by DCs exposed to complement C4.

### Complement C4 promotes T lymphocytes differentiation via DCs in SEL patients

**Fig. 5:**
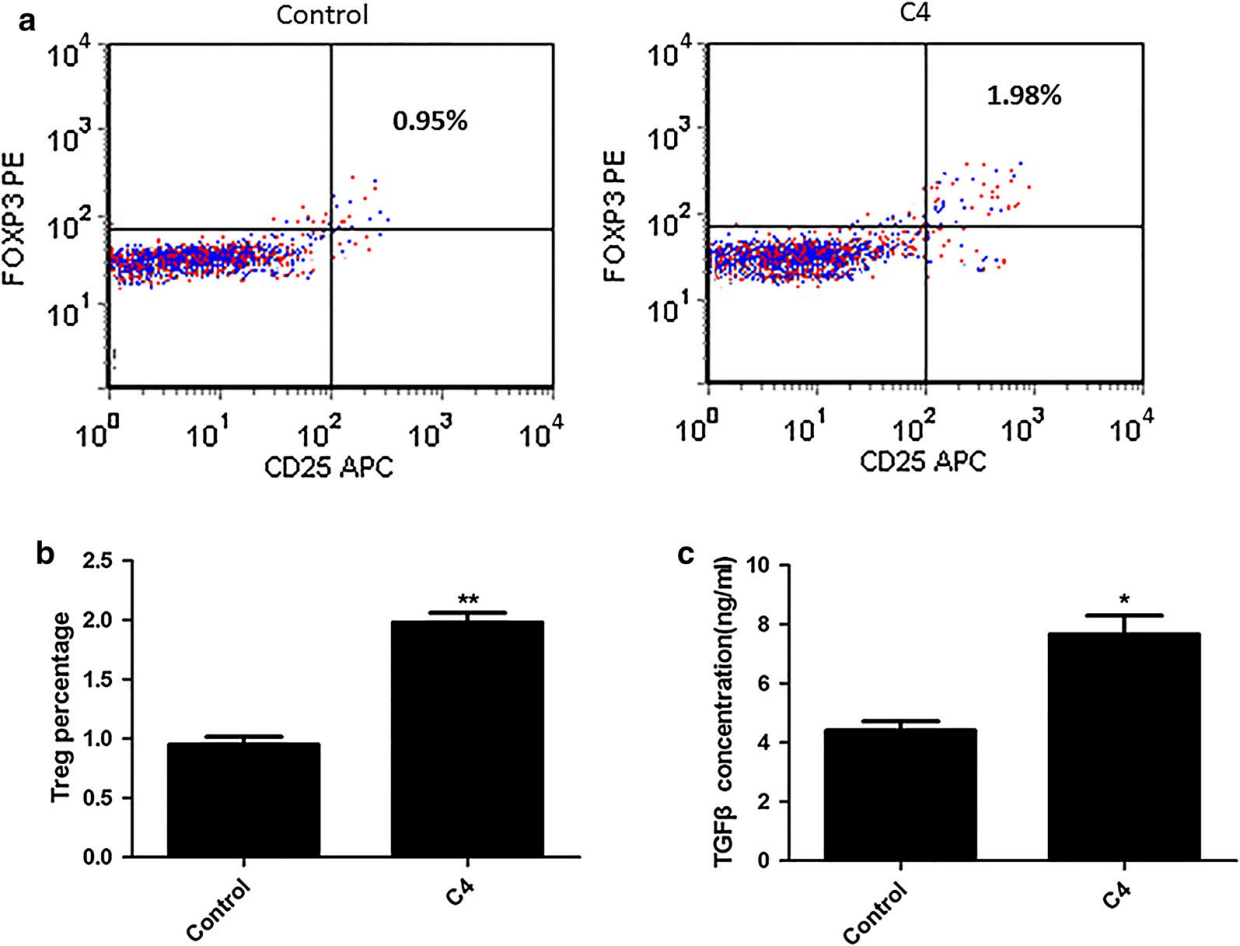
Pretreating DCs from SLE patients with C4 copy numbers <4 with complement C4 promotes T lymphocytes differentiation. Pre-incubated DCs (from SLE patients with C4 copy numbers <4) with 500 μg/ml complement C4 for 48 h, and then incubated with T lymphocytes for 7 days. **a** Flow Cytometer was used to determine the CD4+CD25+FOXP3+Treg percentage; **b** the statistical result of Flow Cytometer was shown; **c** TGFβ concentration of cells was determined by ELISA kit; **p < 0.01 vs control

Isolated DCs from SLE patients with C4 copy numbers <4. DCs were pre-treated with complement C4 before co-incubated with T lymphocytes. It was also observed that complement C4 significantly increased CD4+CD25+FOXP3+Treg cells percentage (Fig. 5a, b) and TGFβ concentration (Fig. 5c) via DCs.

## Discussion

The complement system, exists in tissues and serum, is a part of immune system that helps or complements the ability of antibodies and phagocytic cells to clear pathogens from the organism [17]. C4 is one important component of complement system. The role of complement in SLE is complex. On one hand, complement is integral in the inflammatory reaction in SLE that results in tissue and organ damage. On another hand, the deficiency of complement is a strong risk factor to promote the development of SLE [8, 11, 13, 18]. However, it has been widely considered that complement system plays a protective role against the progression of SLE and other autoimmune disorders in normal subjects. In the present study, we found serum complement C4 concentrations were elevated with the increasing of C4 copy numbers. Waste disposal hypothesis, impaired immune complex processing and dysregulation of cytokines are proposed to explain the causal link between SLE and early classical pathway complement deficiencies [8, 19, 20]. While the exact mechanisms remain to be elucidated. We observed serum complement C4 in SLE patients was positively correlated with Treg percentage. The further studies confirmed that complement C4 modulated T cells differentiation by mediating DCs.

Tregs, commonly known as suppressor T cells, are a component of the immune system that suppress immune responses of other cells and prevent the onset of aberrant self-immune response [21, 22]. Recently, Tregs have been assessed in SEL patients [23–25]. The decreased Tregs number and impaired function are observed in SLE patients [26, 27]. However, it remains unknown whether Tregs are necessary for the pathophysiology of SLE, which is a very important topic for us and other investigators. In this study, the value of Treg percentage was elevated with the increase of serum C4 copy number in patients with SLE, which implied a close relationship between Treg cells and complement C4 level. However, administrated T cells with different concentrations of complement C4, no significant differences were observed in CD4+CD25+FOXP3+Treg population, FOXP3 mRNA and TGFβ concentrations compared with T cells without the treatment of complement C4. FOXP3 appears to function as a master regulator in the development and function of Treg cells [28]. TGFβ is known to be critical for the activation of Treg cells and the expression of FOXP3 [29], and it has been reported that low levels of TGFβ1 correlate with increased SLE disease activity [30]. The above results suggest that complement C4 is associated with Treg cells, but the role of complement C4 on Treg cells is not conducted directly.

There are two ways to have functions on Treg cells by complement, which including direct action and indirect action. William et al. demonstrated that targeting C3a/C3aR and C5a/C5aR interactions could facilitate iTreg-mediated tolerance to alloantigens in humans [31]. Our in vitro study demonstrated that it was not through direct action in C4 induced Treg cells differentiation. Sacks et al. found C5aR activates naïve CD4+ Th cells to undergo differentiation not only to IL-17 producing T helper (Th17) cells but also to Treg via a TGFβ dependent pathway [32]. Recent studies form human and animals provide the evidences for the role of IL-17 and Th17 cells in the pathogenesis of SLE [33, 34]. Therefore, we supposed C4 maybe have effect on promoting Treg cells differentiation via DCs. We found that T cells, co-incubated with DCs and then administrated with complement C4, had a higher level of CD4+CD25+FOXP3+Treg percentage and TGFβ concentration. In addition, DCs pre-treated with complement C4 before co-incubated with T cells, the similar results were observed. These data suggested that DCs took part in mediating T cells differentiation induced by complement C4. Complement receptor type 1 (CR1), the receptor of complement C4, serves as the main system for processing and clearance of complement opsonized immune complexes [35]. Inhibiting CR1 of T cells, which were co-incubated with DCs and then administrated with C4, showed the decreased levels of CD4+CD25+FOXP3+Treg percentage and TGFβ concentration. The data confirmed that C4 mediated the T cells differentiation.

In summary, we found C4 takes part in T cells differentiation to Treg cells via DCs, suggesting that Treg cells serve as the target therapy in preventing or controlling immune-mediated inflammatory disorders. Knowledge about Tregs and C4 and other regulatory cytokines provides new insights in the pathogenesis of SLE. The new lines of investigation for SLE treatment concerning Tregs and C4 will be opened in the near future.
